# Immune Aspects and Myometrial Actions of Progesterone and CRH in Labor

**DOI:** 10.1155/2012/937618

**Published:** 2011-10-19

**Authors:** Nikolaos Vrachnis, Fotodotis M. Malamas, Stavros Sifakis, Panayiotis Tsikouras, Zoe Iliodromiti

**Affiliations:** ^1^2nd Department of Obstetrics and Gynecology, University of Athens Medical School, Aretaieio Hospital, 11528 Athens, Greece; ^2^1st Department of Obstetrics and Gynecology, University of Athens Medical School, Alexandra Hospital, 11528 Athens, Greece; ^3^Department of Obstetrics and Gynecology, University Hospital of Heraklion, 71110 Heraklion, Crete, Greece; ^4^Department of Obstetrics and Gynecology, University Hospital of Alexandroupolis, Democritus University of Thrace, 68100 Alexandroupoli, Greece

## Abstract

Progesterone and corticotropin-releasing hormone (CRH) have a critical role in pregnancy and labor, as changes related to these hormones are crucial for the transition from myometrial quiescence to contractility. The mechanisms related to their effect differ between humans and other species, thus, despite extensive research, many questions remain to be answered regarding their mediation in human labor. Immune responses to progesterone and CRH are important for labor. Progesterone acts as an immunomodulator which controls many immune actions during pregnancy, and its withdrawal releases the inhibitory action on inflammatory pathways. In humans, a “functional” progesterone withdrawal occurs with onset of labor through changes in progesterone metabolism, progesterone receptors, and other molecules that either facilitate or antagonize progesterone function. Placental CRH acts on the fetal pituitary-adrenal axis to stimulate adrenal production of androgens and cortisol and also acts directly on myometrial cells via its receptors. CRH also affects inflammatory signals and vice versa. Interactions between progesterone and CRH additionally occur during labor. We describe the role of these two hormones in human myometrium and their interactions with the immune system during labor.

## 1. Introduction

Mechanical and endocrine mechanisms, immune system responses with inflammatory signals, and release of cytokines, prostaglandins, and oxytocin contribute among others to the transition from myometrial quiescence to labor initiation. The role of these agents has been extensively studied in many animal models, although differences exist between species, and therefore these observations do not always apply to humans [1]. Progesterone and corticotropin-releasing hormone (CRH) are both among the most important mediators of labor. Their role has been studied in many different tissues and organs during pregnancy and labor [2]. Progesterone withdrawal occurs towards the onset of labor through many mechanisms. Placental CRH acts on the fetal pituitary-adrenal axis and directly on myometrial cells to facilitate labor. 

Apart from changes in the endocrine milieu, immune system responses are also vital for labor. During the onset of labor immune cells such as neutrophils, macrophages and T cells invade into cell membranes, decidua, cervix, and myometrium as they are attracted by local chemokines [3, 4]. Characterization of the human myometrial transcriptome and comprehension of the changes in gene expression during spontaneous labor at term have considerably elucidated the association between spontaneous labor and biological processes such as inflammatory response, chemotaxis, and immune response, as well as molecular functions like cytokine and chemokine activity and chemokine receptor binding. Among the overexpressed genes were interleukin 8 (Il-8), Il-6, monocyte chemotactic protein-1 (MCP-1), leukocyte immunoglobulin-like receptor, subfamily A member 5 (LILRA5), chemokine C-C motif ligand 6 (CXCL6), nuclear factor of kappa-light chain gene enhancer in B-cells inhibitor zeta (NFKBIZ), and suppressor of cytokine signaling 3 (SOCS3). Software for pathway analysis of microarrays and gene expression data revealed that the pathways involved in inflammatory response were enriched during spontaneous labor at term [5]. 

The increased expression of cytokines in the myometrium results in increased contractility. Proinflammatory cytokines contribute to the onset of labor irrespectively of the presence of inflammation. Interleukin 1-*β* (IL-1*β*), IL-6, and IL-8 trigger the transcription of genes through activation of nuclear factor kappa-light-chain-enhancer of activated B cells (NF-*κ*B) [6]. IL-6 is considered to be mainly a proinflammatory cytokine [7, 8], but may possibly also have anti-inflammatory properties and its upregulation in parallel with IL-1*β* and tumor necrosis factor-alpha (TNF-*α*) may contribute to a balance between pro- and anti-inflammatory cytokines [9]. Lipoxin A4 is an anti-inflammatory compound that may moderate inflammatory response and modulate inflammatory events taking place in the myometrium during parturition [10]. During labor, myometrial cells are connected by gap junctions that are created by multimers of connexin 43 for the achievement of coordinated and synchronous myometrial contractions, while progestins repress connexin 43 gene expression in myometrial cells [11–13]. Moreover, oxytocin receptors (OTRs) in myometrium increase during labor, and oxytocin promotes myometrial contractility by increasing the intracellular Ca^2+^ ions and the production of prostaglandins [14–16]. In uterine tissues proinflammatory cytokines such as IL-1*β* and TNF increase cyclooxygenase-2 (COX-2) expression, OTRs, Prostaglandin H Synthase type 2 (PGHS-2), and connexin-43 [6]. In this study we review some of the most recent advances in scientific knowledge concerning progesterone and CRH function and interactions in human labor, placing particular focus on the effect on myometrial cells. We also discuss the immune system responses to these hormones.

## 2. Progesterone Withdrawal in Labor

**Figure 1:**
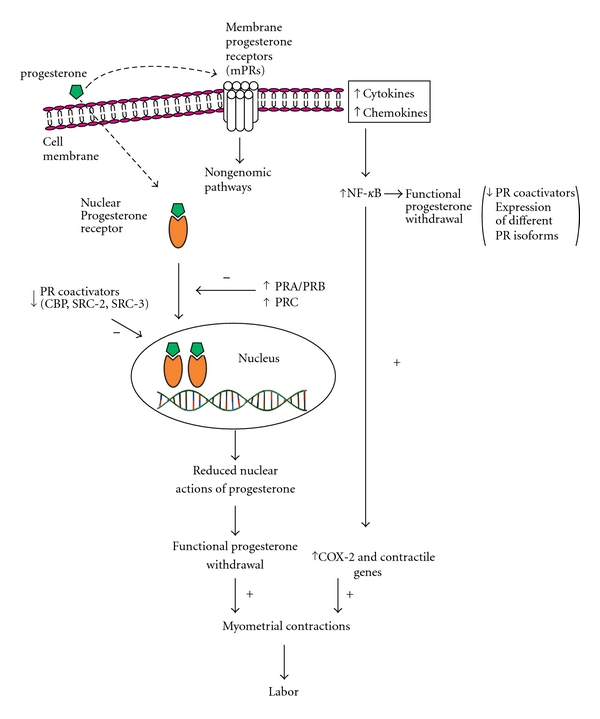
Mechanisms of functional progesterone withdrawal for the initiation of parturition. Progesterone binds to its nuclear receptor PR and activates genomic pathways that maintain uterine quiescence. Functional progesterone withdrawal occurs through many mechanisms: (a) the expression of different PR isoforms (increased PR-A:PR-B ratio and the expression of PR-C), (b) the decreased expression of PR coactivators, such as CBP, SRC-2, SRC-3, (c) binding of progesterone to membrane progesterone receptors (mPR) which activate nongenomic pathways, and (d) immune factors such as cytokines and chemokines activating NF-*κ*B, that in turn lead to functional progesterone withdrawal. NF-*κ*B increases the expression of COX-2 and contractile genes. Progesterone withdrawal together with increased expression of COX-2 and contractile genes results in increased myometrial contractions, which is a vital component for the initiation of labor. PR: progesterone receptor, CBP: CREB-binding protein, SRC: steroid receptor coactivators, NF-*κ*B: nuclear factor kappa-light-chain enhancer of activated B cells, mPRs: membrane progesterone receptors, COX-2: cyclooxygenase-2.

According to the “progesterone block” hypothesis, proposed by Arpad Csapo, progesterone blocks myometrial contraction and maintains pregnancy, while its withdrawal transforms the myometrium to the laboring state [17]. In rats and other animals the initiation of labor coincides with a decline in progesterone serum concentration. However, in humans progesterone levels remain high throughout pregnancy and during labor [1]. This has led to the hypothesis of a “functional” progesterone withdrawal that may occur through mechanisms such as progesterone metabolism into inactive forms, expression of different progesterone receptor isoforms, altered expression of molecules that act as progesterone coregulators, and finally the antagonism of NF-*κ*B [18] with progesterone (Figure 1). 

### 2.1. Progesterone Metabolism

In mice, the catabolism of progesterone to the inactive C21-steroid 5*α* dihydroprogesterone by the enzyme steroid 5*α*-reductase facilitates cervical ripening, while mice deficient in this enzyme fail to deliver at term despite normal uterine contractions and progesterone withdrawal in blood [19]. In mice with a targeted disruption of 20*α*-hydroxysteroid dehydrogenase (20*α*-HSD) gene (which converts progesterone to the biologically inactive metabolite 20*α*-dihydroprogesterone), the mean duration of pregnancy is significantly prolonged compared to the controls [20]. In cultured rat ovarian granulose cells IL-1*β* stimulates 20*α*-hydroxysteroid dehydrogenase activity [21]; however it is unknown if such an effect may also take place in myometrium during labor. In humans, experiments with endocervical cells of women in labor show that, during cervical ripening and parturition, 17*β*-hydproxysteroid dehydrogenase type 2 activity decreases, resulting in increased 20*α*-hydroxyprogesterone and estradiol levels because of decreased conversion of 20*α* hydroxyrogesterone to progesterone and decreased conversion of estradiol to estrone. These changes result in inactivation of progesterone responses [22].

### 2.2. Altered Expression of Progesterone Receptors

The role of progesterone receptors in human pregnancy has been recently extensively reviewed [23]. In humans, there are two major isoforms of progesterone receptor, PR-A and PR-B, which belong to the nuclear receptor superfamily, as well as many other isoforms which have so far given evidence of being less significant. The expression of the various isoforms may contribute to the functional withdrawal of progesterone during labor. In human myometrial cells, the ratio of PR-A:PR-B mRNA increases 2- to 3-fold compared with the nonlaboring state, mainly due to overexpression of PR-A. This change induces a “functional estrogen activation” through increased estrogen receptor *α* (ER*α*) expression [24]. PR-A may also suppress the transcriptional activity of PR-B, which is the main receptor for the nuclear signal transduction of progesterone [25, 26]. Apart from myometrial contractions, the functional progesterone withdrawal due to the altered expression of PR-A, PR-B isoforms may also contribute to the cervical changes during labor [27, 28]. 

The binding of progesterone to PR-C, which is a soluble form of the receptor, may sequester available progesterone away from PR-B and thereby diminish its biological effect [18]. In another study of women in labor, PR-C protein levels were increased in cytoplasmic fractions of fundal myometrial cells, but not in cells of the lower uterine segment, while PR-B protein levels increased only in the laboring fundal endometrium. In the same study PR-A could not be detected despite the presence of PR-A mRNA. This paradoxical increase in PR-B protein may be the result of the associated reduced progesterone activity. In human telomerase reverse transcriptase- (hTERT-) immortalized human myometrial cells, PR-C compromises PR-B transactivation. The upregulation of PR-B and PR-C expression was associated with activation of NF-*κ*B, while upregulation of NF-*κ*B was observed in pregnant mouse uterus, coinciding with PR isoform expression. In addition, treatment of myometrial cells with the cytokine IL-1*β* further induces NF-*κ*B activation and concomitant increase in PR expression, which suggests that labor-associated changes in myometrium are associated with uterine inflammatory pathways [29].

A number of other progesterone receptors (mPR-*α*, mPR-*β*, and mPR-*γ* ) have been identified which are believed to be structurally related to the G-protein complex. mPR-*α* and mPR-*β* receptors were found to be expressed in human pregnant myometrial cells, and progesterone activation of these receptors may induce nongenomic actions, resulting in inhibition of adenyl cyclase, phosphorylation of myosin light chains, and contractions of human myometrial cells in labor. It is suggested that mPR at the end of pregnancy downregulates steroid receptor coactivator-2 (SRC-2), which in combination with an altered PR-B:PR-A ratio may lead to decreased transcriptional activation of PR-B [30]. Controversy exists regarding the function of mPRs on cell membrane, some authors supporting the view that the isoforms of these receptors are intracellular and reside primarily on the endoplasmic reticulum, while progesterone does not induce any G protein-dependent signaling of mPRs [31].

### 2.3. Altered Expression of PR Coregulators

PRs interact with coregulators that either increase (coactivators) or decrease (corepressors) their transcriptional activity. These molecules include among others the CREB-binding protein (CBP) and the steroid receptor coactivators SRC-1, SRC-2, and SRC-3. In fundal myometrial cells of women in labor, the mRNA levels of SRC-2, SRC-3, and CBP are decreased compared to the nonlabor samples. Nuclear levels of CBP, SRC-2, and SRC-3 proteins were also reduced in fundal myometrium during labor. In the mouse uterus in labor, levels of SRC-1, SRC-3, and CBP were also reduced. The reduced levels of these coactivators were related to decreased acetylated histone H3 in both human and mouse, suggesting the closing of chromatin structure and the reduced expression of PR-responsive genes as a possible mechanism for the initiation of labor [32]. Histone acetylation/deacetylation can also affect NF-*κ*B activity and proinflammatory gene expression (e.g., IL-6, IL-8, COX-2, and RANTES), while histone deacetylase inhibitors exert anti-inflammatory effects in myometrium which may favor uterine quiescence [33]. 

p54nrb (non-POU-domain-containing, octamer binding protein) is another molecule that acts as a PR transcriptional corepressor in vitro and which decreases in rat myometrium at term pregnancies. Its decreased expression at the time of labor may contribute to the increased expression of labor-associated genes [34].

### 2.4. Antagonistic Action of NF-*κ*B

An antagonism between NF-*κ*B and progesterone exists in reproductive tissues during labor. NF-*κ*B belongs to the superclass 4 of transcriptional factors and is involved in the synthesis of many proinflammatory mediators such as cytokines and COX-2. Inflammation is a critical trigger for the initiation of term and preterm labor [35], and NF-*κ*B as a transcriptional factor is implicated in proinflammatory stimuli signaling. Proinflammatory cytokines such as IL-1*β* and TNF-*α*, as well as lipopolysaccharides (LPSs), can stimulate NF-*κ*B activity in the uterus. Additionally, the promoter region of cytokines genes such as IL-1*β*, IL-8, and IL-6 contains NF-*κ*B recognition elements, and NF-*κ*B promotes the synthesis of cytokines [36]. 

 NF-*κ*B also stimulates prostaglandin synthesis, regulates matrix metalloproteinase expression, connexin-43 (a gap junction protein), oxytocin receptor, and may express inhibitory PR isoforms or increase progesterone metabolism [18, 36]. In lower segment fibroblasts and in amnion cells IL-1*β* induces the synthesis of NF-*κ*B regulated genes, while progesterone attenuates these effects [37]. Progesterone exhibits anti-inflammatory properties that maintain uterine quiescence. MCP-1 is a chemokine which is produced by decidual, endometrial, myometrial, and trophoblast cells. It is increased in laboring pregnant myometrium prior to the onset of labor and attracts leukocytes from the circulation to the myometrium. MCP-1 is also increased in cultured myometrial cells in response to IL-1*β*. Experimentally, in choriodecidual cells MCP-1 expression is suppressed by progesterone, while, by contrast, it is stimulated by NF-*κ*B [38–40].

Antagonism between nuclear factors such as NF-*κ*B and PR for nuclear cofactors is another proposed mechanism of functional progesterone withdrawal. During labor, the level of NF-*κ*B changes in the intracellular environment, and its activity is regulated by interactions with the nuclear cofactors that increase gene expression, such as CBP/p300. The antagonism between NF-*κ*B and PR for CBP may contribute to progesterone withdrawal and expression of labor-promoting genes [41].

### 2.5. Progesterone as an Immunomodulator in Pregnancy and the Antagonism with Cytokines

Progesterone acts as an immunomodulator that interacts with the immune system and exerts anti-inflammatory effects throughout pregnancy. Progesterone inhibits the activity of dendritic cells (DCs) that generate proinflammatory responses and favor the induction of tolerogenic DCs. It also controls the activity of natural killer (NK) cells and the differentiation of T cells into T-helper cell type 2 (Th2) like clones. The Th2 phenotype induced by progesterone is a prerequisite for the maintenance of pregnancy [42, 43]. 

Progesterone-induced blocking factor (PIBF) is a protein which is released from lymphocytes in response to progesterone, mediates the immunological effects, and induces the production of Th2 dominant cytokines like IL-3, IL-4, and IL-10 [44]. The functional progesterone withdrawal and the attenuation of progesterone's effects may contribute, among other factors, to the swift from a Th2 to a Th1 dominant effect towards labor. Inflammatory response involves Toll-like receptors, and progesterone modulates the expression of these receptors in mouse cervix and placenta [45]. 

Cytokines such as IL-1*β* and TNF-*α* increase PGHS-2 and therefore the synthesis of prostaglandins (PG), while they downregulate prostaglandin 15-hydroxy dehydrogenase (PGDH), the enzyme that converts PGs into inactive metabolites. On the contrary, progesterone promotes PGDH expression [6]. Progesterone also downregulates prostaglandin F2-alpha receptor, which is increased by IL-1*β* [46]. Via the use of expression microarray and quantitative reverse transcriptase PCR it has been shown that in human myometrial cells medroxyprogesterone acetate, a synthetic progestogen, affects genes involved in inflammatory response and cytokine activity. Among the downregulated genes of the study were those of IL-1*β*, Il-6, Il-11, Il-24, COX-2, and connexin 43 [47]. 

 Clinically, women with threatened preterm labor have significantly lower serum concentrations of PIBF and IL-10 and significant higher serum concentrations of the proinflammatory cytokines, IL-6 and interferon-*γ* (IFN-*γ*), compared with healthy pregnant women of the control group [48].

### 2.6. The Effect of Progesterone Withdrawal on ZEB 1, 2 and miRNAs 

A novel pathway for progesterone withdrawal and increased expression of contraction-associated genes has recently been described based on experiments in mice and human endometrial cells. The levels of micro-RNAs (mi-RNAs), a class of posttranscriptional regulators that belong to the mi-RNA-200 family, increase with advanced gestation, as do the levels of connexin-43 and oxytocin receptor, while the levels of ZEB1 and ZEB2 (zinc finger E-box binding homeobox proteins 1 and 2) repressor transcriptional factor decline. ZEB1 and ZEB2 inhibit the expression of connexin-43 and oxytocin receptor and block the oxytocin-induced contraction of myometrial cells. ZEB1 and ZEB2 also inhibit the expression of the miRNA-200 family, which in turn downregulate ZEB1, ZEB2. Progesterone increases the levels of ZEB1, and thus progesterone withdrawal reverses the inhibitory effect of ZEB on mi-RNAs. As mi-RNAs increase, they cause repression of ZEB and thereby promote the synthesis of connexin-43 and oxytocin receptor, thus facilitating labor [49].

In summary, all the above described mechanisms may collectively impair progesterone regulation of gene expression, and this functional progesterone withdrawal promotes uterine contractility, effecting labor.

### 2.7. Clinical Applications of Progesterone in Human Labor

Progesterone has clinical applications with regard to either the initiation of labor or the arrest of preterm labor. In women, treatment with antiprogestins induces labor and delivery, but the most significant applications concern prevention of preterm birth. Numerous studies and meta-analyses have been conducted to evaluate the efficacy of progesterone in reducing the incidence and complications of preterm labor. Progestational agents may reduce the number of preterm labor and their complications in certain cases, but the optimal route of administration, the optimal dose, and the frequency of administration have not been clearly defined [50–54].

## 3. Corticotropin-Releasing Hormone Function on Myometrial Contractility

CRH levels in plasma rise exponentially in human pregnancy. However, this increase is more rapid in women who deliver preterm and slower in women who deliver postterm, compared to women who deliver at term. This rise begins early in pregnancy (16–20 weeks) and provides evidence that placenta acts through CRH as a “clock” that controls the length of pregnancy. Moreover, preterm birth may not be just the result of an event around the time of delivery but a process that begins from early in pregnancy [55]. 

### 3.1. Functions of Placental CRH

The rise of CRH is associated with a concomitant fall in CRH binding protein (CRHBP) in late pregnancy. The binding of CRH to CRHBP makes it biologically inactive and removes it from circulation. The decrease in CRHBP during the final three weeks of pregnancy results in higher concentrations of active CRH in circulation that contribute to the onset of labor [55, 56]. In both term and preterm labor, the expression of CRH mRNA from the placenta is increased, with this increase being higher in preterm deliveries. However, placental CRHBP mRNA expression does not change despite the fact that CRHBP decreases in maternal circulation before labor. This leads to the hypothesis that another source of CRHBP exists, such as a fetal source that may be responsible for this decrease [55, 57].

**Figure 2:**
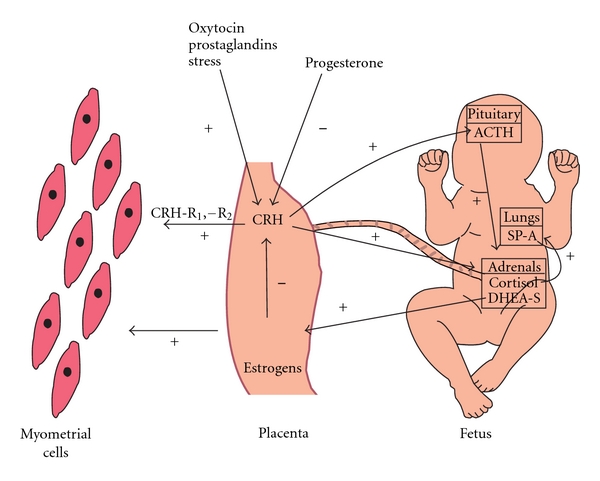
The role of CRH in human labor. CRH is produced in the placenta in response to stimuli that either increase (oxytocin, PG, stress) or decrease (progesterone) its production. CRH acts on fetal pituitary to increase ACTH production. ACTH and CRH act on fetal adrenals that produce DHEA-S and cortisol. DHEA-S is metabolized in the placenta to estrogens that increase myometrial contractions and facilitate labor. Cortisol acts on fetal lungs which produce SP-A, which also increases uterine contractility. Furthermore, placental CRH acts directly on human myometrial cells via its receptors CRH-R1, -R2, to facilitate the transition from uterine quiescence to myometrial contractions during labor. CRH: corticotrophin-releasing hormone, PG: prostaglandins, ACTH: adrenocorticotropic hormone, DHEA-S: dehydroepiandrosterone sulphate, SP-A: surfactant protein A.

However, despite the fact that the levels of CRH in maternal circulation rise by as much as 1000 times compared to the nonpregnant state, only a mild hypercortisolism occurs, mainly due to estrogen-induced production of cortisol-binding protein, and the maternal axis continues its function. Thus the functional target of placental CRH is not the maternal pituitary adrenal axis but the fetal pituitary adrenal axis [56]. Placental CRH stimulates adrenocorticotropic hormone (ACTH) production from the fetal pituitary. ACTH stimulates fetal adrenals to produce dehydroepiandrosterone (DHEA), dehydroepiandrosterone-sulphate (DHEA-S), and cortisol. ACTH is also produced in the placenta through paracrine mechanisms. Placental CRH also exerts a direct effect by stimulating fetal adrenal zone cells (Figure 2). Fetal adrenal DHEA is metabolized to estrogens in the placenta that favor parturition [2, 58, 59]. The produced cortisol exerts a stimulatory effect on the placenta to further produce CRH, thus a positive loop is established that causes placental CRH to rise exponentially as pregnancy advances. Estrogens, progesterone, and nitric oxide inhibit CRH production, while a number of neuropeptides exert a stimulatory effect [2]. Cytokines influence CRH production. IL-1*β* and TNF as well as lipopolysaccharides activate the hypothalamic-pituitary-adrenal axis and induce CRH production, while CRH regulates IL-1*β*, IL-6, and lipopolysaccharides production by immune cells [60]. Cytokines also influence the metabolism of cortisol. The enzyme 11*β*-hydroxysteroid dehydrogenase (11*β*-HSD) converts cortisol to the inactive metabolite cortisone. Cytokines such as IL-1*β*, IL-6, and TNF-*α* inhibit human placenta 11*β*-HSD type 2 isoform, and this could result in increased cortisol levels and therefore increased CRH production by the placenta [6]. 

Cortisol produced by fetal adrenals acts on fetal lungs and produce surfactant protein A (SP-A) that activates inflammatory signals in the uterus, which consequently enhance myometrial contractility [18]. SP-A signals via Toll-like receptors. These are membrane-spanning receptors that act as key regulators in immune system responses [61]. SP-A exerts its action via activation of NF-*κ*B in many cells. In the mouse, SP-A increases the production of IL-1*β* and NF-*κ*B by amniotic fluid macrophages and causes migration of macrophages in the uterus, resulting in the activation of the inflammatory cascade [62]. 

CRH may also facilitate the expression of contraction-associated genes. In myometrial smooth muscle cells from nonpregnant women, CRH enhances connexin-43 mRNA and protein expression through nuclear transcription factor activator protein 1 (AP-1) activation and upregulation of c-Fos expression, which is an AP-1 subunit. This is accomplished in a positive CRH-dose-dependent manner. As a result, in late pregnancy the high levels of CRH possibly cause an associated increased expression of connexin-43 that promote myometrial contractions [63]. AP-1 also increases gene expression in response to many other stimuli, including cytokines [64]. CRH may also increase the myometrial response to prostaglandin F2*α* and therefore modulate the onset of labor [65].

### 3.2. The Role of Myometrial CRH Receptor Isoforms in Labor

CRH exerts its actions through activation of specific receptors, termed CRH-R1 and CRH-R2. These receptors belong to the family of seven transmembrane (7TMD) G protein coupled receptors (GPCRs). CRH receptors are expressed in several tissues in pregnancy. Both CRH-R1 and CRH-R2 and their variants have been detected in human myometrium, and it is believed that the expression of various isoforms plays a role in both myometrium quiescence and transition to a contractile phenotype during labor [66]. Alternative splicing of CRH-R mRNA results in different receptor isoforms (such as CRH-R1*α* and CRH-R1d) with different activities that generate a more contractile phenotype [67]. In myometrial strips from laboring and nonlaboring pregnant women, it was shown that CRH could inhibit spontaneous contractility of nonlaboring but not of laboring myometrium, acting via the CRH-R1 receptor. This effect further implies that CRH may exhibit a dual role at the myometrium [68]. 

It is suggested that CRH-R1 promotes relaxation of smooth muscles via intracellular signals that inhibit phosphorylation of the contractile protein myosin light chain (MLC20). The decreased expression of proteins such as G*α*_s_-protein (a G protein subunit that activates the cAMP-dependent pathway by activating adenylate cyclase) towards the end of pregnancy turns the balance towards myometrial contractility. The increased expression of CRH-R2 protein toward onset of labor increases MLC20 phosphorylation and myometrial contractility [67]. 

CRH-R1 and CRH-R2 have been identified both in the upper and lower uterine segment of laboring and nonlaboring human myometrium, but during labor CRH-R1 decreases significantly at the upper segment but not at the lower one. This decreased expression may be related to the fact that during labor the fundus of the uterus switches to a highly contractile state, while the lower segment remains relatively quiescent [69]. Recently a new isoform of CRH-R has been identified in human pregnant but not in nonpregnant myometrium. This isoform is termed CRH-R1*β*/d and shows similarities with both CRH-R1*β* and -R1d, such as reduced affinity for CRH, negligible signaling, and mainly cytoplasmic localization. Its expression is maximum at the early third trimester, and it is increased by estradiol-17*β* [70].

### 3.3. The Effect of Urocortins and Interleukins on CRH Receptors

CRH receptors may also be activated by other agonists such as the urocortins. Urocortin 2 (UCN 2) is a CRH-R agonist that is expressed in human pregnant myometrium and interacts with CRH-R2 receptors and through a signal transduction pathway involving the protein kinase C (PKC), the extracellular signal-regulated kinases ERK1/2, and the RhoA/ROK. Urocortin 2 interaction stimulates MLC20 phosphorylation, which in turn induces myometrial contractility [71]. Although urocortins 2 and 3 are expressed in the human placenta, it is not known if they act by increasing myometrial contractility during labor [72]. Recent data have reported that, in human placentas from women with PPROM and chorioamnionitis, inflammatory events are associated with changes to the CRH-related mechanism of labor, such as increased CRH, UCN 2, and CRH-R1 mRNA expression and decreased UCN 3 and CRH-R2 expression [73].

Immune system responses are also associated with changes to the CRH-related mechanisms of labor. Experiments on both term and preterm, laboring and nonlaboring human myometrium show that active labor is associated with increase of CRH-R1*α* and CRH-R1*β* mRNA in term and preterm myometrium. IL-1*β* induces CRH-R1_ T_ *α* mRNA and prostaglandin synthetase 2 (PTGS2) mRNA and protein levels, decreases CRH-R1*β* mRNA, and impairs CRH-induced cAMP production. It may therefore be hypothesized that IL-1*β* acts as a regulator of CRH-R1 expression and function that contributes to a preparatory contractile environment necessary for the initiation of labor [74]. 

CRH may also act on both term and preterm cervical fibroblasts and increase IL-8 expression, which is an important mediator of cervical ripening [75].

### 3.4. Clinical Applications of CRH in Human Labor 

Several blood, amniotic fluid, or vaginal fluid markers have been proposed as predictors of preterm labor, including interleukins, cortisol, fibronectin, ADAM-8, ITAC, estriol, a-FP, b-HCG, and others [76–81]. Many of these studies have evaluated the clinical usefulness of CRH measurements to predict preterm labor [56, 82, 83]. Urocortin may also serve as a biomarker of labor in women with threatened preterm birth before 34 weeks [84]. However, these observations have mostly diagnostic and not therapeutic benefit, since no CRH/CRH receptor antagonist has so far been identified that can be used in humans to prevent preterm labor.

## 4. Interactions between Progesterone and CRH in Labor

In human placental cells progesterone inhibits CRH production, but this effect is modulated differently from PR-A and PR-B, since PR-A overexpression leads to decreased CRH promoter activity, while PR-B increases CRH promoter activity in the presence of exogenous progesterone [85]. Progesterone induces CRH-R1 and CRH-R1*β* variants, which lead to increased action of CRH, effecting under these conditions uterine quiescence during pregnancy. The effect of functional progesterone withdrawal on these receptors may enable myometrial contractions during labor [70]. CRH causes relaxation of term and preterm human myometria, and progesterone acts synergistically to enhance it in term but not in preterm myometrium. This provides extra evidence that progesterone withdrawal in late pregnancy may lessen the relaxant effect of CRH [86].

CRH may also itself contribute to progesterone withdrawal towards labor. Experiments in human placental trophoblasts show that CRH decreases placental production of progesterone by reducing the levels of the progesterone synthesis enzymes cytochrome 450 CYP11A1 and HSD3B1. In contrast, treatment with a CRH antagonist increases progesterone production. This effect of CRH on progesterone production is mediated through a PKC-dependent pathway [87].

## 5. Conclusion

Progesterone is a highly important hormone that contributes to the maintenance of pregnancy. Progesterone withdrawal occurs either by decreasing plasma levels in animals or by functional withdrawal mechanisms in humans. CRH is another significant hormone which, like progesterone, acts via different mechanisms either to support pregnancy or to facilitate the process of labor. These two hormones interact with each other to promote myometrial quiescence or contractility. Inflammatory immune responses such as infiltration of uterus with immune cells and production of cytokines contribute significantly to the mechanisms of labor. Progesterone and CRH regulate some of these immune actions. Despite intensive research for many years, there are still a large number of enigmas and areas of controversy regarding human labor and the role of both hormones and inflammation. Animal models do not show similar mechanisms to those of the human fetus and this is one of the obstacles to attaining better understanding of human labor. In the future, clinical applications based on the actions of progesterone, CRH and inflammatory immune responses may be essential for the prevention of preterm labor, which is today a major cause of neonatal morbidity and mortality.
